# Conservative versus Invasive Strategy in Elderly Patients with Non-ST-Elevation Myocardial Infarction: Insights from the International POPular Age Registry

**DOI:** 10.3390/jcm12175450

**Published:** 2023-08-22

**Authors:** Wout W. A. van den Broek, Marieke E. Gimbel, Dean R. P. P. Chan Pin Yin, Jaouad Azzahhafi, Renicus S. Hermanides, Craig Runnett, Robert F. Storey, David Austin, Rohit Oemrawsingh, Justin Cooke, Gavin Galasko, Ronald J. Walhout, Dirk A. A. M. Schellings, Stijn L. Brinckman, Hong Kie The, Martin G. Stoel, Antonius A. C. M. Heestermans, Debby Nicastia, Mireille E. Emans, Arnoud W. J. van ’t Hof, Hannes Alber, Robert Gerber, Paul F. M. M. van Bergen, Ismail Aksoy, Abdul Nasser, Paul Knaapen, Cees-Joost Botman, Anho Liem, Johannes C. Kelder, Jurriën M. ten Berg

**Affiliations:** 1 Department of Cardiology, St. Antonius Hospital, Koekoekslaan 1, 3435 CM Nieuwegein, The Netherlands; mariekegimbel@gmail.com (M.E.G.); d.chanpinyin@antoniusziekenhuis.nl (D.R.P.P.C.P.Y.); j.azzahhafi@antoniusziekenhuis.nl (J.A.); keld01@antoniusziekenhuis.nl (J.C.K.); j.ten.berg@antoniusziekenhuis.nl (J.M.t.B.); 2 Department of Cardiology, Isala Hospital, Dokter van Heesweg 2, 8025 AB Zwolle, The Netherlands; r.s.hermanides@isala.nl; 3 Department of Cardiology, Northumbria Healthcare NHS Foundation Trust, 8 Silver Fox Way, Newcastle upon Tyne NE27 0QJ, UK; craig.runnett@northumbria-healthcare.nhs.uk; 4 Department of Infection, Immunity and Cardiovascular Disease, University of Sheffield, Sheffield S10 2TN, UK; r.f.storey@sheffield.ac.uk; 5 Academic Cardiovascular Unit, The James Cook University Hospital, Marton Rd., Middlesbrough TS4 3BW, UK; david.austin@nhs.net; 6 Department of Cardiology, Albert Schweitzer Hospital, Albert Schweitzerplaats 25, 3318 AT Dordrecht, The Netherlands; r.m.oemrawsingh@asz.nl; 7 Department of Cardiology, Chesterfield Royal Hospital NHS Foundation Trust, Chesterfield Rd., Calow, Chesterfield S44 5BL, UK; justincooke@nhs.net; 8 Department of Cardiology, Blackpool Teaching Hospital NHS Foundation Trust, Whinney Heys Rd., Blackpool FY3 8NR, UK; dr.galasko@nhs.net; 9 Department of Cardiology, Gelderse Vallei Hospital, Willy Brandtlaan 10, 6716 RP Ede, The Netherlands; walhoutr@zgv.nl; 10 Department of Cardiology, Slingeland Hospital, Kruisbergseweg 25, 7009 BL Doetinchem, The Netherlands; d.schellings@slingeland.nl; 11 Department of Cardiology, Tergooi MC, Rijksstraatweg 1, 1261 AN Blaricum, The Netherlands; sbrinckman@tergooi.nl; 12 Department of Cardiology, Treant Zorggroep, Boermarkeweg 60, 7824 AA Emmen, The Netherlands; s.the@treant.nl; 13 Department of Cardiology, Medisch Spectrum Twente, Koningstraat 1, 7512 KZ Enschede, The Netherlands; m.stoel@mst.nl; 14 Department of Cardiology, Noordwest Hospital Group, Wilhelminalaan 12, 1815 JD Alkmaar, The Netherlands; a.a.c.m.heestermans@mca.nl; 15 Department of Cardiology, Gelre Hospital, Albert Schweitzerlaan 31, 7334 DZ Apeldoorn, The Netherlands; d.nicastia@gelre.nl; 16 Department of Cardiology, Ikazia Hospital, Montessoriweg 1, 3083 AN Rotterdam, The Netherlands; memans@cardiologieopzuid.nl; 17 Department of Cardiology, Maastricht University Medical Center, P. Debyelaan 25, 6229 HX Maastricht, The Netherlands; arnoud.vant.hof@mumc.nl; 18 Cardiovascular Research Institute Maastricht, Universiteitssingel 50, 6229 ER Maastricht, The Netherlands; 19 Department of Cardiology, Zuyderland Medical Centre, Henri Dunantstraat 5, 6419 PC Heerlen, The Netherlands; 20 Department for Internal Medicine and Cardiology, KABEG Klinikum, Feschnigstraße 11, 9020 Klagenfurt am Wörthersee, Austria; hannes.alber@kabeg.at; 21 Department of Cardiology, East Sussex Healthcare NHS Foundation Trust, Dane Rd., Seaford BN25 1DH, UK; r.gerber@nhs.net; 22 Department of Cardiology, Dijklander Hospital, Maelsonstraat 3, 1624 NP Hoorn, The Netherlands; 23 Department of Cardiology, Admiraal de Ruyter Hospital, ‘s-Gravenpolderseweg 114, 4462 RA Goes, The Netherlands; i.aksoy@adrz.nl; 24 Department of Cardiology, South Tyneside and Sunderland NHS Foundation Trust, Harton Ln., South Shields NE34 0PL, UK; abdulnasser@nhs.net; 25 Department of Cardiology, Amsterdam University Medical Centre, De Boelelaan 1117, 1081 HV Amsterdam, The Netherlands; p.knaapen@vumc.nl; 26 Department of Cardiology, Sint Jans Gasthuis, Vogelsbleek 5, 6001 BE Weert, The Netherlands; cjbmbotman@onsbrabantnet.nl; 27 Department of Cardiology, Franciscus Gasthuis, Kleiweg 500, 3045 PM Rotterdam, The Netherlands; liemanho@gmail.com

**Keywords:** coronary artery disease, non-ST-elevation myocardial infarction, elderly, conservative strategy, invasive strategy

## Abstract

This registry assessed the impact of conservative and invasive strategies on major adverse clinical events (MACE) in elderly patients with non-ST-elevation myocardial infarction (NSTEMI). Patients aged ≥75 years with NSTEMI were prospectively registered from European centers and followed up for one year. Outcomes were compared between conservative and invasive groups in the overall population and a propensity score-matched (PSM) cohort. MACE included cardiovascular death, acute coronary syndrome, and stroke. The study included 1190 patients (median age 80 years, 43% female). CAG was performed in 67% (N = 798), with two-thirds undergoing revascularization. Conservatively treated patients had higher baseline risk. After propensity score matching, 319 patient pairs were successfully matched. MACE occurred more frequently in the conservative group (total population 20% vs. 12%, _adj_HR 0.53, 95% CI 0.37–0.77, *p* = 0.001), remaining significant in the PSM cohort (18% vs. 12%, _adj_HR 0.50, 95% CI 0.31–0.81, *p* = 0.004). In conclusion, an early invasive strategy was associated with benefits over conservative management in elderly patients with NSTEMI. Risk factors associated with ischemia and bleeding should guide strategy selection rather than solely relying on age.

## 1. Introduction

As life expectancy is advancing and the prevalence of coronary artery disease increases with age, the number of elderly patients with coronary artery disease (CAD) is rising [1,2]. Elderly patients with non-ST-elevation myocardial infarction (NSTEMI) are at higher risk of cardiovascular events, as well as treatment-related complications (especially bleeding) [3]. Moreover, as frailty is common, these patients are rarely included in clinical trials, which is why guidelines are often based on the extrapolation of data from a substantially younger and healthier population or studies with a small sample size. Due to this insufficient evidence, both the European Society of Cardiology (ESC) and American Heart Association/American College of Cardiology (AHA/ACC) can only provide valuable considerations but do not give specific recommendations for the treatment of elderly patients with an NSTEMI [4,5,6]. In practice, elderly patients less often receive guideline-recommended care, despite current guidelines recommending treating older patients with the same interventional strategies as younger patients [4,7].

The POPular AGE Registry was initiated in order to specifically capture the clinical treatment strategy and prognosis of this heterogeneous population. Further aims were to evaluate differences in cardiovascular risk between a conservative and invasive strategy and find predictors for major adverse cardiovascular events (MACE).

## 2. Materials and Methods

The POPular AGE registry is an investigator-initiated, prospective, observational, international, multicenter study of patients ≥75 years presenting with NSTEMI. There were no exclusion criteria. Patients were recruited between 1st August 2016 and December 2019 at 29 sites in the Netherlands, the United Kingdom and Austria. Decisions regarding medical therapy, the performance of invasive coronary angiography (CAG) and, if indicated, subsequent percutaneous coronary intervention (PCI) or coronary artery bypass grafting (CABG) were at the discretion of the attending physicians, except for a number of patients (N = 111) who were also enrolled in the POPular AGE trial and were randomized between ticagrelor and clopidogrel [8]. To assess differences in baseline characteristics and outcomes in different treatment strategies, the population was divided into two treatment groups: the invasive group, defined as patients who underwent CAG during the index hospitalization, and a conservative group receiving medical therapy alone. The invasive strategy was further stratified to CAG only, PCI or CABG. This study was conducted according to the principles of the Declaration of Helsinki and was approved by the Medical Research Ethics Committees United (ID: W17.021). All patients provided written informed consent.

Demographic, clinical and procedural characteristics and in-hospital and one-year follow-up data were collected. At one and twelve months, patients were sent a questionnaire inquiring about current medication use, events and quality of life by use of the Short Form Health Survey 12 (SF-12) and new hospital admissions. Frailty was assessed within one month after admission using the Groningen Frailty Indicator (GFI) (Appendix A). Cardiovascular events consisted of all-cause death, cardiovascular death, recurrent acute coronary syndrome (ACS), stroke, transient ischemic attack (TIA) and bleeding (Bleeding Academic Research Consortium [BARC] criteria) at one-year follow-up [9]. ACS included ST-segment elevation myocardial infarction (STEMI), NSTEMI, unstable angina pectoris, and type 2 myocardial infarction (MI), defined using the Fourth Definition of MI [10]. MACE was defined as a composite of cardiovascular death, ACS and stroke. Net adverse clinical events (NACE) were defined as a composite of all-cause death, ACS, definite stent thrombosis, stroke or Bleeding Academic Research Consortium (BARC 3 or 5) bleeding. Major bleeding was defined as a bleeding event of BARC type 3 or 5, and major or clinically relevant nonmajor bleeding was defined as a bleeding event of BARC type 2, 3 or 5. Antithrombotic therapy at discharge consisted of a P2Y12-inihibitor with aspirin or oral anticoagulation (OAC), or triple therapy consisting of aspirin, a P2Y12-inhibitor and OAC. Optimal medical treatment (OMT) was defined according to the guideline as the use of a beta-blocker, ACE-inhibitor or angiotensin II receptor blocker, and statin in combination with adequate antithrombotic therapy.

Continuous variables are presented as mean ± standard deviation (SD) or as median with interquartile range (IQR); categorical variables are presented as frequencies and percentages. Differences in baseline characteristics and events during follow-up between the invasive and conservative groups were tested with chi-square or Fisher exact tests for categorical variables and two-sample t-test or Mann-Whitney U test for continuous variables. Estimation of the incidence of the ischemic and bleeding endpoints was carried out using the Kaplan-Meier method. Hazard ratios (HR) with 95% confidence intervals (CI) were calculated using Cox proportional-hazard models. Violation of the proportional hazards assumption was evaluated by calculating Schoenfeld residuals. To adjust for possible confounders, clinically relevant variables or characteristics that differed at baseline were selected for univariate regression analysis (Table S1). If there was a significant interaction in the univariate analysis, they were selected for multivariate regression analysis. Only those characteristics with a significant interaction in the multivariate analysis were included in the final model.

Propensity scores were estimated using a multivariable logistic regression model, with the invasive strategy as the dependent variable. Covariates were chosen based on clinical relevance and their relation with the treatment strategy or the clinical outcome, or both, as assessed using a regression model. The covariates included in the matching process were age, gender, diabetes mellitus, hypercholesterolemia, hypertension, smoking, previous PCI, previous MI, Killip class, left ventricular ejection fraction below 50%, ST-depression, peripheral artery disease, heart failure, chronic kidney disease, chronic obstructive pulmonary disease (COPD) and optimal medical treatment (OMT). Propensity score matching was performed using a one-to-one matching protocol without replacement (greedy-matching algorithm) within a caliper equal to 0.2 of the standard deviation of the logit of the propensity score. To assess the effect of frailty and bleeding risk on clinical outcomes, we performed sub-group analyses in frail patients (known frailty score of 4 or higher) and patients with high bleeding risk (HBR) (CRUSADE score of 40 or higher) [9]. Missing baseline covariates were imputed using the median before the propensity score model was finalized. Standardized mean differences of more than 0.10 were considered as evidence of imbalance. All tests were two-tailed and used a *p*-value < 0.05 to characterize statistical significance. All analyses were performed using R statistical software version 4.1.2.

## 3. Results

### 3.1. Baseline Characteristics

**Figure 1:**
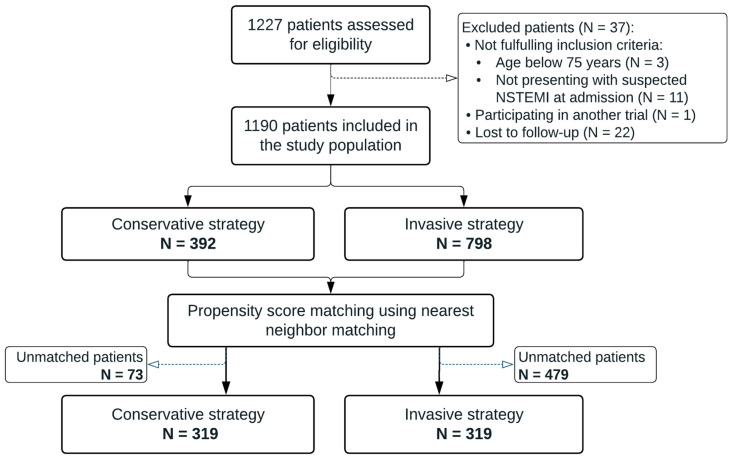
Flowchart of the study. NSTEMI = non-ST-elevation myocardial infarction.

Between 1 August 2016 and 23 December 2019, a total of 1227 patients were enrolled in the registry. Fourteen patients did not meet the eligibility criteria, twenty-two patients were lost to follow-up, and one patient was enrolled in another clinical trial. The final population consisted of 1190 patients (Figure 1). The baseline characteristics of the study population are presented in Table 1. The median age was 80 (IQR 77–84) years and 43% of the population was female (N = 507). At discharge, 90% of patients were diagnosed with NSTEMI. The remaining patients had unstable angina pectoris (5%), non-specific chest pain (3%), ST-elevation MI (1%), Takotsubo cardiomyopathy (<1%), pericarditis (<1%) or exacerbation of COPD (<1%). Overall missing data at baseline was low, except for GRACE risk score (15%), Body Mass Index (26%), left ventricular ejection fraction (LVEF) at baseline (37%) and CRUSADE bleeding score (38%).

#### 3.1.1. Treatment

The median duration of hospital admission was 5 days (IQR 3–9). During the index hospital admission, CAG was performed in 67% of all patients (N = 798), mostly by radial artery access (72%) (N = 568). Significant coronary artery disease (>50% diameter reduction) was demonstrated in 85% of the cases (N = 676). Of patients undergoing CAG, 52% (N = 418) were subsequently treated with PCI, 14% (N = 111) with CABG and the remaining 34% (N = 270) only with medical treatment, of which one-third (N = 90) had no significant coronary artery disease (Table S2). On average, patients who underwent CAG were younger, more often male and had more risk factors for coronary artery disease (e.g., hypercholesterolemia, history of smoking) than patients in the conservative group (Table 1). After PSM, 319 pairs of patients were successfully matched. There was no significant difference in baseline characteristics between the two groups after PSM regarding the covariates used in the PSM (Table 1). Standardized differences were less than 0.10 for all covariates used in the PSM analysis, indicating that there was no evidence of imbalance between the groups (Figures S1 and S2).

#### 3.1.2. Antithrombotic Therapy

At discharge, DAPT was the most commonly prescribed antithrombotic treatment (55%, N = 655) (Table S3). In patients receiving a P2Y12-inhibitor agent at discharge (N = 925, 480 with clopidogrel and 439 with ticagrelor), early discontinuation or switching of P2Y12-inhibitor occurred in 15% (N = 138). For both clopidogrel and ticagrelor, the most important reasons for early discontinuation or switching were undergoing CABG, bleeding and concomitant use of OAC. Of 74 patients who discontinued ticagrelor, 16 (22%) did so because of dyspnea. Early discontinuation was comparable within the invasive and conservative groups.

#### 3.1.3. Frailty and Quality of Life

Frailty data at 1 month was available in 898 patients (Table 2). Of these patients, 60% (N = 541) had a GFI score of 4 or higher indicating frailty. Self-reported frailty was less common in the invasive group than in the conservative group (56% vs. 70%, *p* <0.001) (Table 2). The average quality of life within one month after admission was 37.1 ± 6.1 for the physical component summary (PCS) and 44.5 ± 6.0 for the mental component summary (MCS) of the SF-12 questionnaire. After PSM, there were no statically significant differences regarding frailty (68% vs. 62%, *p* = 0.224) and quality of life (PCS at 1 month: 36.4 vs. 37.0, *p* = 339, MCS at 1 month: 43.8 vs. 44.5, *p* = 0.256) between the conservative and invasive groups.

### 3.2. Outcomes

#### 3.2.1. Total Population

After one year, MACE occurred in 14% of patients (N = 171) in the total population. NACE occurred in one in four patients (25%, N = 295). The total bleeding rate was 13% (N = 153), of which clinically relevant minor and major bleeding occurred in 11% of patients (N = 135). In the multivariable analysis, age (HR 1.05, 95% CI 1.02–1.09, *p* = 0.001), diabetes mellitus (HR 1.59, 95% CI 1.15–2.22, *p* = 0.006), Killip class of 2 or higher (HR 1.53, 95% CI 1.04–2.27, *p* = 0.033), LVEF below 50% (HR 1.47, 95% CI 1.00–2.15, *p* = 0.049) and ST-depression (HR 1.66, 95% CI 1.19–2.33, *p* = 0.003) were independent predictors for MACE at baseline (Table 3).

**Figure 2:**
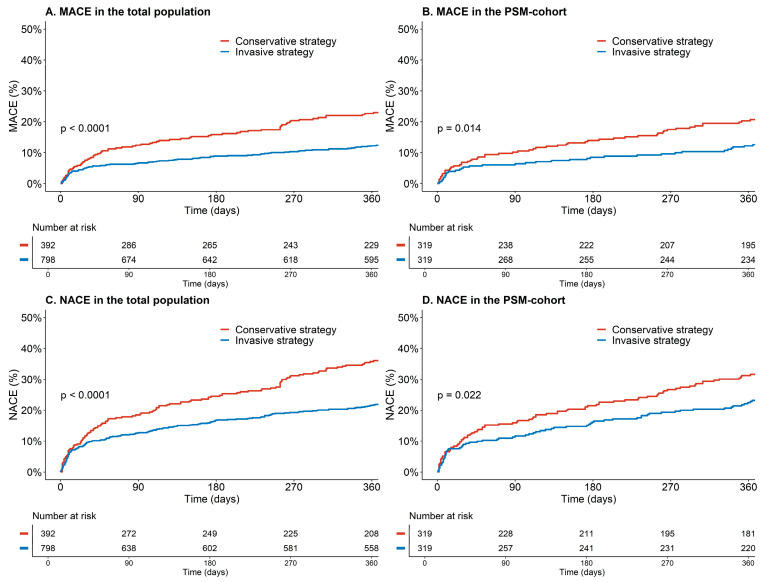
Kaplan Meier Curve for major adverse cardiovascular events (MACE) and Net Adverse Clinical Events (NACE) in the invasive and conservative groups. (**A**) Kaplan Meier Curve for MACE and Net Adverse Clinical Events (NACE) in the total population before propensity score matching. (**B**) Kaplan Meier Curve for MACE after propensity score matching. (**C**) Kaplan Meier Curve for NACE in the total population before propensity score matching. (**D**) Kaplan Meier Curve for NACE after propensity score matching.

**Figure 3:**
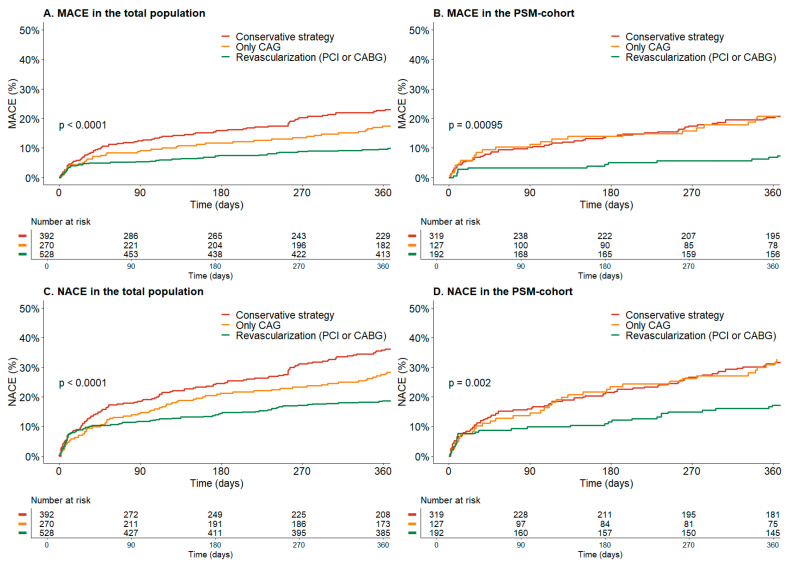
Kaplan Meier Curve for major adverse cardiovascular events (MACE) and Net Adverse Clinical Events (NACE) between the different treatment strategies; conservative treatment, only coronary angiography (CAG) or revascularization with percutaneous coronary intervention (PCI) or coronary artery bypass grafting (CABG). (**A**) Kaplan Meier Curve for MACE in the total population before propensity score matching. (**B**) Kaplan Meier Curve for MACE after propensity score matching. (**C**) Kaplan Meier Curve for NACE in the total population before propensity score matching. (**D**) Kaplan Meier Curve for NACE after propensity score matching.

In the total population, the invasive strategy was associated with a lower risk for MACE after multivariable adjustment (12% vs. 20%, _adj_HR 0.53, 95% CI 0.37–0.77, *p* = 0.001) (Table 4 and Figure 2A). Undergoing revascularization with PCI or CABG was associated with an even lower risk of MACE after multivariable adjustment (_adj_HR0.39, 95% CI 0.26–0.61, *p* =< 0.001) (Figure 3A). There was a significant difference in clinically relevant major and minor bleeding (14% vs. 6%, _adj_HR 1.85, 95% CI 1.11–3.10, *p* = 0.012) between the invasive and conservative groups. Major bleeding was numerically higher, but not statistically significantly different (6% vs. 2%, _adj_HR 2.24, 95% CI 0.96–5.22, *p* = 0.062). In the invasive group, periprocedural bleeding occurred in 3% of patients (N = 27).

#### 3.2.2. PSM Cohort

After PSM, an invasive strategy remained associated with a lower risk for MACE compared to a conservative strategy (_adj_HR 0.50, 95% CI 0.31–0.81, *p* = 0.004) (Table 4 and Figure 2B). Similarly, revascularization with either PCI or CABG was associated with an even lower risk for MACE after PSM (_adj_HR 0.39, 95% CI 0.27–0.61, *p* < 0.001). An invasive strategy continued to demonstrate a significant decrease in MACE in the subgroup of patients aged 80 years or older (_adj_HR 0.54, 95% CI 0.31–0.92, *p* = 0.024). Bleeding rates for both major bleeding and non-major and clinically relevant bleeding remained higher in the invasive group (Table 4). However, NACE was still lower in the invasive group. This holds true for both the entire cohort (33% vs. 21%, HR 0.65, 95% CI 0.49–0.86, *p* = 0.003) and the PSM cohort (29% vs. 22%, HR 0.69, 95% CI 0.48–0.99, *p* = 0.045). Since differences in the use of aspirin, P2Y12 inhibitors and cholesterol inhibitors persisted after PSM, we conducted a sensitivity analysis with additional adjustments for these variables, which showed consistent results.

#### 3.2.3. Frailty

Frail patients (N = 541) who underwent an invasive strategy had a lower incidence of MACE (9.1% vs. 18.1%, _adj_HR 0.49, 95% CI: 0.30–0.82, *p* = 0.006) and NACE (18.7% vs. 28.2%, _adj_HR 0.53, 95% CI 0.34–0.84, *p* = 0.007) than non-frail patients, but a higher rate of clinically relevant major and minor bleeding (13.9% vs. 5.9%, _adj_HR 2.29, 95% CI 1.19–4.42, *p* = 0.013). Results were consistent in the PSM cohort.

#### 3.2.4. High Bleeding Risk

In HBR patients (N = 240), an invasive strategy reduced the risk for MACE (17.5% vs. 32.5%, _adj_HR 0.40, 95% CI 0.22–0.74, *p* = 0.003), while increasing the risk for clinically relevant major and minor bleeding (16.2% vs. 2.5%, _adj_HR 5.21, 95% CI 1.23–22.09, *p* = 0.025). However, in non-HBR patients (N = 496), the risk for both MACE (10.9% vs. 12.9%, _adj_HR 0.86, 95% CI 0.40–1.85, *p* = 0.70) and clinically relevant major and minor bleeding (14.8% vs. 12.9%, _adj_HR 0.85, 95% CI 0.44–1.64, *p* = 0.63) was comparable in both groups. The analysis yielded similar results in the PSM cohort.

## 4. Discussion

In this large, international, prospective, observational registry, we evaluated the treatment and survival of patients aged 75 years or older with NSTEMI. The main findings were (1) lower-risk patients were more likely to undergo CAG. (2) Age, diabetes mellitus, reduced LVEF below 50%, Killip class of 2 or higher and ST-depression at admission were independent predictors for MACE. (3) Patients who were conservatively treated had a higher risk of MACE than patients who underwent an invasive strategy, while undergoing revascularization was associated with an even lower risk of MACE, with robust results after propensity score analysis.

Treating elderly patients with NSTEMI is challenging. They more often present with atypical symptoms, and are a heterogeneous group with multiple comorbidities, variable frailty and functional status [11]. These patients are also at high risk for cardiovascular events, therefore, it is of utmost importance to perform adequate risk assessment when opting for invasive treatment. The identified predictors for MACE may assist in estimating this risk. Our data also suggest that although age is associated with a worse prognosis, older age alone should not be a reason to opt for a conservative strategy.

### 4.1. Invasive versus Conservative Strategy

The 2020 ESC Guidelines for the management of ACS in patients presenting without persistent ST-segment elevation recommends applying the same interventional strategies in older patients as for younger patients [4]. Unfortunately, these recommendations are largely based on studies in which older patients were underrepresented, studies with small sample sizes or highly selected populations [12,13,14,15]. Observational data has shown that an invasive strategy lowers the risk of all-cause death in elderly NSTEMI patients [16,17,18]. In these reports, conservatively treated patients were, as in our population, older and had more cardiovascular co-morbidities. It is highly plausible that these differences are due to indication bias, as physicians are less keen to invasively treat older and frail patients, probably reinforced by the fact that older patients also have a higher risk of procedure-related complications [19]. This has been described before as the risk-treatment paradox, meaning that the benefit of revascularization increases with cardiovascular risk, yet an increased cardiovascular risk results in those patients not being revascularized [20,21]. Randomization can overcome this paradox. The After Eighty study randomized a highly selected population of 457 patients with NSTEMI ≥80 years to an invasive or conservative strategy, as only 11% of eligible patients were finally included in the trial [13]. An invasive strategy was superior to the conservative strategy regarding the composite of myocardial infarction, need for urgent revascularization, stroke and death (HR 0.53, 95% CI 0.41–0.69). The findings in the PSM cohort are consistent with those of the After Eighty study, as evidenced by similar hazard ratios, particularly in the subgroup analysis comprising patients aged 80 years and above (_adj_HR 0.54, 95% CI 0.31–0.92). A meta-analysis, which incorporated the findings of the After Eighty study along with two smaller prematurely terminated RCTs, revealed similar results with a significant reduction in recurrent MI and urgent revascularization, but no survival benefit [11,22,23,24]. In contrast to the aforementioned studies, we observed a reduction in all-cause death and cardiovascular death. This difference may be due to unmeasured confounding and the selective indication for the invasive treatment of patients in healthier conditions. Ongoing randomized trials, such as the SENIOR-RITA trial (NCT03052036), will further refine existing evidence and contribute to solutions for this clinical dilemma.

Noteworthy is the notable difference in the Kaplan Meier curves between patients who underwent CAG without revascularization and those who were conservatively treated, before and after PSM (Figure 3C,D). This suggests that actual revascularization is necessary for the best clinical outcome. However, it should be noted that this difference may also be influenced by selection bias, as it is possible that more patients who only underwent CAG were diagnosed with type 2 MI, which has been associated with a higher mortality rate than type 1 MI in most studies [25].

As opposed to the After Eighty study, we did observe an increase in bleeding events in the invasive group, which can partly be explained by the difference in antithrombotic treatment and less use of the radial access for PCI leading to higher periprocedural bleeding event rates. Throughout the enrolment period, the prevailing standard of care in the invasive group was a 12-month duration of dual antiplatelet therapy (DAPT). This likely contributed to the elevated rates of bleeding observed, highlighting the importance of the recommendations in the current guidelines advocating for a shorter DAPT duration in patients at high risk of bleeding, for which age above 75 years is an important criterion [4]. Reducing the bleeding risk, especially in HBR patients, is an important issue that should be addressed, as bleeding events have a negative impact on prognosis and quality of life [26,27]. Therefore, it is important to carefully assess one’s risk of periprocedural and bleeding complications before opting for an invasive strategy. Our subgroup analysis revealed that effectively utilizing the CRUSADE score differentiated patients who were at high risk of bleeding in the invasive group. This highlights the role of clinical risk scores in facilitating risk stratification and treatment decisions.

### 4.2. Frailty and Quality of Life

Self-reported frailty and quality of life were assessed by the use of questionnaires at 1 and 12 months. The GFI score was missing in 25% of patients and SF-12 scores were missing in more than 40%. We deliberately chose to survey the questionnaires at one month, in order to prevent the hospitalization from influencing the score. Therefore, we did not use these data in our Cox regression models. The available data suggested that frailty was more common in patients in the conservative group. Quality of life also appeared to be worse in the conservative group. Clinical decision-making during hospitalization was probably partly based on functional status and frailty and, therefore, may explain these differences to some extent. This may have played a role in the difference in outcome between the conservative and invasive groups. However, when assessing frailty and quality of life after PSM, we saw no difference between the conservative and invasive groups. In frail patients, an invasive strategy remained the most beneficial treatment strategy based on the net clinical benefit, implying that an invasive strategy remains valuable in these patients. However, it should be noted that the assessment of frailty was based on questionnaires administered one month after hospitalization, which may limit the robustness of our results and warrants further investigation.

### 4.3. Strengths and Limitations

This registry is a large cohort study of elderly patients with NSTEMI in the Netherlands, the United Kingdom and Austria. Both academic and non-academic centers participated in this registry, making our data representative of NSTEMI patients in routine clinical practice. We made use of propensity score analyses to adjust for differences in patient characteristics, which is a well-accepted statistical methodology for the purpose of comparing non-randomized patient cohorts [28]. However, there were some limitations to our study. The first is its observational design. It is still probable that, despite using PSM, our results are subject to selection bias, which may account for the observed difference in risk and survival between the invasive and conservative groups. Similarly, unmeasured confounding may have influenced the treatment strategy and survival. Second, despite using questionnaires to assess frailty and quality of life, data regarding functional status and neuropsychiatric symptoms were limited. Third, the completeness of revascularization and reasons for clinical decision-making between the conservative and invasive groups were not registered. Finally, the presence of missing data might have biased the results.

## 5. Conclusions

In this prospective registry of real-world NSTEMI patients of 75 years or older, MACE and major bleeding were frequent. We found age, diabetes mellitus, reduced LVEF, Killip class and ST-depression at admission as independent predictors for MACE. In a population of elderly patients with NSTEMI, opting for an early invasive strategy was associated with benefits over conservative management. When deciding on the most suitable approach, it is essential to consider risk factors related to both ischemia and bleeding, rather than solely relying on age as the sole determining factor.

## Figures and Tables

**Table 1:** Baseline characteristics of the total study population before and after propensity score matching.

	Before PSM			After PSM		
	Conservative (N = 392)	Invasive (N = 798)	*p*-Value	Conservative (N = 319)	Invasive (N = 319)	*p*-Value
Age (years)—median, IQR	83 (79–87)	79 (77–83)	<0.001	82 (79–86)	82 (79–85.5)	0.757
Female	199 (51%)	308 (39%)	<0.001	156 (49%)	140 (44%)	0.204
BMI (mean ± SD)	27 ± 5	27 ± 4	0.688	27 ± 5	27 ± 4	0.127
**Medical history**						
MI	143 (36%)	227 (29%)	0.005	107 (34%)	108 (34%)	0.933
Stroke	69 (18%)	111 (14%)	0.095	58 (18%)	57 (18%)	0.918
PAD	30 (8%)	94 (12%)	0.029	26 (8%)	29 (9%)	0.672
Heart failure	48 (12%)	31 (4%)	<0.001	21 (7%)	26 (28%)	0.449
CKD	70 (18%)	66 (8%)	<0.001	38 (12%)	41 (13%)	0.718
COPD	65 (17%)	87 (11%)	0.006	43 (14%)	48 (15%)	0.571
Atrial fibrillation	94 (24%)	112 (14%)	<0.001	70 (22%)	60 (19%)	0.326
Dyslipidemia	129 (33%)	370 (46%)	<0.001	109 (34%)	122 (38%)	0.284
Diabetes mellitus	116 (30%)	191 (24%)	0.036	91 (29%)	97 (30%)	0.602
Hypertension	241 (62%)	529 (66%)	0.103	197 (62%)	206 (65%)	0.460
Smoking	149 (38%)	367 (46%)	0.009	126 (40%)	135 (42%)	0.469
**At admission**						
Killip class > II	76 (19%)	91 (11%)	<0.001	53 (17%)	53 (17%)	1.000
LVEF < 50%	73 (19%)	142 (18%)	0.727	55 (17%)	59 (19%)	0.679
ST-depression	117 (30%)	240 (30%)	0.936	94 (30%)	100 (31%)	0.606
GRACE-score (mean ± SD)	165 ± 41	161 ± 41	0.117	163 ± 40	165 ± 44	0.565
CRUSADE score (mean ± SD)	40 ± 12	34 ± 11	<0.001	39 ± 12	37 ± 12	0.088
**Treatment**						
Optimal medical treatment	110 (28%)	364 (46%)	<0.001	101 (32%)	110 (35%)	0.449
Aspirin	220 (56%)	638 (80%)	<0.001	181 (57%)	237 (74%)	<0.001
P2Y12-inhibitor	265 (68%)	662 (83%)	<0.001	222 (70%)	247 (77%)	0.025
Beta-blocker	254 (65%)	607 (76%)	<0.001	215 (67%)	223 (70%)	0.495
ACE-inhibitor	151 (39%)	418 (52%)	<0.001	132 (41%)	146 (46%)	0.264
AT-II antagonist	58 (15%)	154 (19%)	0.056	49 (15%)	62 (19%)	0.175
Cholesterol inhibitor	261 (67%)	694 (87%)	<0.001	218 (68%)	259 (81%)	<0.001

Data are n (%) unless stated otherwise. ACE-inhibitor = angiotensin-converting-enzyme inhibitor; AT-II = angiotensin-II; CKD = chronic kidney disease; COPD = chronic obstructive pulmonary disease; IQR = interquartile range; LVEF = left ventricular ejection fraction; MI = myocardial infarction; PAD = peripheral arterial disease; PSM = propensity score matching.

**Table 2:** Frailty and quality of life outcomes.

Questionnaire	Number of Patients (Conservative vs. Invasive)	Total Population	Conservative Group	Invasive Group	*p*-Value
GFI—median, IQR	898 (357 vs. 541)	4 (2–7)	6 (3–8)	4 (2–6)	<0.001
Frailty (GFI ≥ 4)		541 (60%)	188 (70%)	353 (56%)	<0.001
SF-12 at 1 month—mean ± SD PCS MCS	728 (232 vs. 496)	37.1 ±6.1 44.5 ±6.0	36.1 ± 6.6 43.4 ± 6.2	37.6 ± 5.7 44.9 ± 5.9	0.002 0.002
SF-12 at 12 months—mean ± SD PCS MCS	591 (150 vs. 441)	37.8 ± 5.8 45.7 ± 5.5	36.4 ± 6.4 44.3 ± 6.2	38.3 ± 5.4 46.2 ± 5.1	0.001 0.001

Data are n (%) or mean ± SD. GFI = Groningen Frailty Indicator; IQR = interquartile range; PCS = physical component summary; MCS = mental component summary, SD = standard deviation. SF-12 = Short-Form 12.

**Table 3:** Univariable and multivariable Cox Regression Analysis for Major Adverse Cardiovascular Events.

Variables	Univariable Model			Multivariable Model		
	HR	95% CI of HR	*p*-Value	HR	95% CI of HR	*p*-Value
Age	1.06	1.03–1.09	<0.001	1.05	1.02–1.09	0.001
Diabetes Mellitus	1.60	1.16–2.22	0.004	1.59	1.15–2.22	0.006
Killip Class of 2 or higher	1.94	1.32–2.84	0.001	1.53	1.04–2.27	0.033
LVEF < 50%	1.70	1.17–2.45	0.005	1.47	1.00–2.15	0.049
ST-depression	1.89	1.35–2.61	<0.001	1.66	1.19–2.33	0.003

CI = confidence interval; HR = hazard ratio; LVEF = left ventricular ejection fraction.

**Table 4:** Cardiovascular outcomes before and after propensity score matching.

	Total Study Population				After PSM			
	Conservative (N = 392)	Invasive (N = 798)	HR * (95% CI)	*p*-Value	Conservative (N = 319)	Invasive (N = 319)	HR (95% CI)	*p*-Value
MACE	78 (20%)	93 (12%)	0.53 (0.37–0.77)	0.001	57 (18%)	38 (12%)	0.50 (0.31–0.81)	0.004
NACE	128 (33%)	167 (21%)	0.65 (0.49–0.86)	0.003	91 (29%)	70 (22%)	0.69 (0.48–0.99)	0.045
All-cause death	100 (26%)	70 (9%)	0.36 (0.25–0.52)	<0.001	70 (22%)	37 (12%)	0.46 (0.29–0.74)	0.001
CV death	49 (13%)	32 (4%)	0.35 (0.21–0.60)	<0.001	36 (11%)	19 (6%)	0.47 (0.24–0.90)	0.023
Recurrent ACS	32 (8%)	49 (6%)	0.47 (0.28–0.78)	0.004	22 (7%)	16 (5%)	0.56 (0.27–1.17)	0.123
Stroke	1 (0.3%)	11 (1%)	2.62 (0.31–22.3)	0.378	1 (0.3%)	0		
Major bleeding	7 (2%)	48 (6%)	2.24 (0.96–5.22)	0.062	4 (1%)	24 (8%)	4.37 (1.50–12.8)	0.007
Non-major and clinically relevant bleeding	22 (6%)	113 (14%)	1.94 (1.16–3.23)	0.012	17 (5%)	53 (17%)	2.52 (1.40–4.51)	0.002
Periprocedural bleeding	0	27 (3%)	NA	NA	0	13 (4%)	NA	NA

* Hazard ratios in the total study population were adjusted for multiple baseline characteristics (Table S1). Data are n (%). ACS = acute coronary syndrome; CV = cardiovascular; MACE = Major Adverse Cardiac events; NACE = Net Adverse Clinical Events.

## Data Availability

The data underlying this article will be shared on reasonable request to the corresponding author.

## Supplementary Materials

The following supporting information can be downloaded at: https://www.mdpi.com/article/10.3390/jcm12175450/s1, Figure S1: Distribution of propensity scores; Figure S2: Covariate balance; Table S1: Cox regression models; Table S2: Coronary lesions; Table S3: Medical therapy at discharge.

## Appendix A

### Appendix A.1. The Groningen Frailty Questionnaire

The Groningen Frailty Questionnaire measures frailty by the use of self-assessment questionnaire. It uses 15 questions measuring the dimensions of physical and psychosocial vulnerability. Eight items have two response categories (yes/no), six items have three response categories (yes/sometimes/no), and one item has a Likert response category (1–10). All items were dichotomized to calculate GFI sum scores. A higher GFI sum score indicates a greater level of frailty, with a maximum score of 15. Higher scores indicate higher frailty levels. A person is considered to be frail when the GFI sum score is 4 points or higher.

### Appendix A.2. SF-12

The SF-12 is a self-reported outcome measure assessing the impact of health on an individual’s everyday life, used to measure quality of life. It consists of 12 items, measuring eight concepts: physical functioning, role limitations due to physical health problems, bodily pain, general health, vitality, social functioning, role limitations due to emotional problems and mental health. The SF-12 can be used to calculate the Physical Component Summary (PCS) and Mental Component Summary (MCS) scales. The average score for an average population is 50 points, with a standard deviation of 10 points. A score below 50 points will therefore indicate a lower quality of life compared to an average population.
