# Supplementary material for: Conservative versus Invasive Strategy in Elderly Patients with Non-ST-Elevation Myocardial Infarction: Insights from the International POPular Age Registry
Source: J Clin Med. 2023 Aug 22;12(17):5450. doi: 10.3390/jcm12175450 (PMC10487667; doi:10.3390/jcm12175450)
Supplement: Supplementary file 1 [file jcm-12-05450-s001.zip › jcm-2524670-Supplementary.pdf]

Table S1. Univariate and Multivariate Cox regression models for adjustment for confounders for all cardiovascular outcomes. Differences in outcomes between conservative-management and invasive-management were calculated using Cox proportional-hazard models. Multiple clinically relevant baseline variables were analyzed in an univariate regression analysis. Variables with a p-value <0.05 were assessed in a multivariate model. The final model included only those characteristics with a significant interaction (p < 0.05) in the multivariate analysis.

Endpoints	Variables tested in univariate regression	Significant variables in multivariate regression model
MACE	Age, gender, prior MI, HF, CKD, AF, DM, COPD, HT, history of smoking, hypercholesterolemia, positive family history, LVEF <50%, Killip class >2, ST-depression, treatment with P2Y12-inhibitors, treatment with OAC	Age, LVEF <50%, Killip class >2, DM, ST-depression
NACE		Age, CKD, Killip class >2, DM, ST-depression
All-cause death		Age, LVEF <50%, Killip class >2
CV death		Age, LVEF <50%, Killip class >2
Recurrent ACS		Diabetes, hypercholesterolemia
Stroke		Peripheral artery disease, hypertension
BARC 3 or 5		-
BARC 2, 3 or 5		HT

ACS = acute coronary syndrome; AF = atrial fibrillation; BARC = Bleeding Academic Research Consortium; COPD = chronic obstructive pulmonary disease; CV = cardiovascular death; DM = diabetes mellitus; HF = heart failure; HT = hypertension; LVEF = left ventricular ejection fraction; MACE = major adverse cardiovascular events; MI = myocardial infarction; NACE = Net adverse clinical events; OAC = oral anticoagulation.

Table S2. Coronary lesions across the different treatment groups.

Vessel disease	OMT (N = 392)	Only CAG (N = 270)	PCI with stent (N = 417)	CABG (N = 111)
No VD		90 (33%)	0	0
1-VD		48 (18%)	123 (30%)	5 (5%)
2-VD		32 (12%)	113 (27%)	20 (18%)
3-VD		73 (27%)	104 (25%)	74 (67%)
Graft dysfunction		22 (8%)	21 (5%)	2 (2%)
VD unknown	392 (100%)	5 (2%)	52 (13%)	10 (9%)

CABG = coronary artery bypass grafting; CAG = coronary angiography; OMT = optimal medical therapy; PCI = percutaneous coronary intervention; VD = vessel disease.

Table S3. Medical therapy at discharge

	**Total (N = 1190)**	**Conservative group (N = 392)**	**Invasive group (N = 798)**
Triple therapy	77 (6%)	10 (3%)	67 (8%)
DAPT*	655 (55%)	157 (42%)	415 (59%)
DAPT with clopidogrel	219 (38%)	77 (49%)	142 (34%)
DAPT with ticagrelor	348 (60%)	80 (51%)	268 (65%)
$P2Y_{12}$ inhibitor+(N)OAC	124 (10%)	54 (14%)	70 (9%)
Aspirin+(N)OAC	33 (3%)	8 (2%)	25 (3%)
Monotherapy aspirin	93 (8%)	36 (9%)	57 (7%)
Monotherapy $P2Y_{12}$ inhibitor	71 (6%)	35 (9%)	36 (5%)
Monotherapy (N)OAC	41 (3%)	18 (5%)	23 (3%)
No antithrombotic therapy	11 (<1%)	6 (2%)	5 (<1%)
Beta-blocker	861 (72%)	254 (66%)	607 (76%)
ACE-inhibitor	569 (48%)	151 (39%)	418 (52%)
Angiotensin II inhibitor	212 (18%)	58 (15%)	154(19%)
Calcium antagonist	306 (26%)	80 (21%)	226 (28%)
Cholesterol inhibitor	955 (80%)	261 (67%)	694 (87%)
Diuretics	365 (31%)	139 (36%)	226 (28%)
Anti-diabetics	245 (21%)	84 (22%)	161 (20%)
Aldosterone antagonist	96 (8%)	39 (10%)	57 (7%)
PPI	888 (75%)	246 (63%)	642 (81%)

Data are n (%). Triple therapy consists of aspirin, a P2Y12 inhibitor and a (non-vitamin-k) oral anticoagulant; ACE = Angiotensin-converting enzyme; DAPT = dual antiplatelet therapy consisting of aspirin and a P2Y12 inhibitor; (N)OAC = (non-vitamin-k-antagonist) oral anticoagulant; PPI = proton pump inhibitor. *In this analysis, patients who were also included in the POPular AGE trial were excluded (total population, N = 1079, of which 372 and 702 in the conservative and invasive group, respectively.)

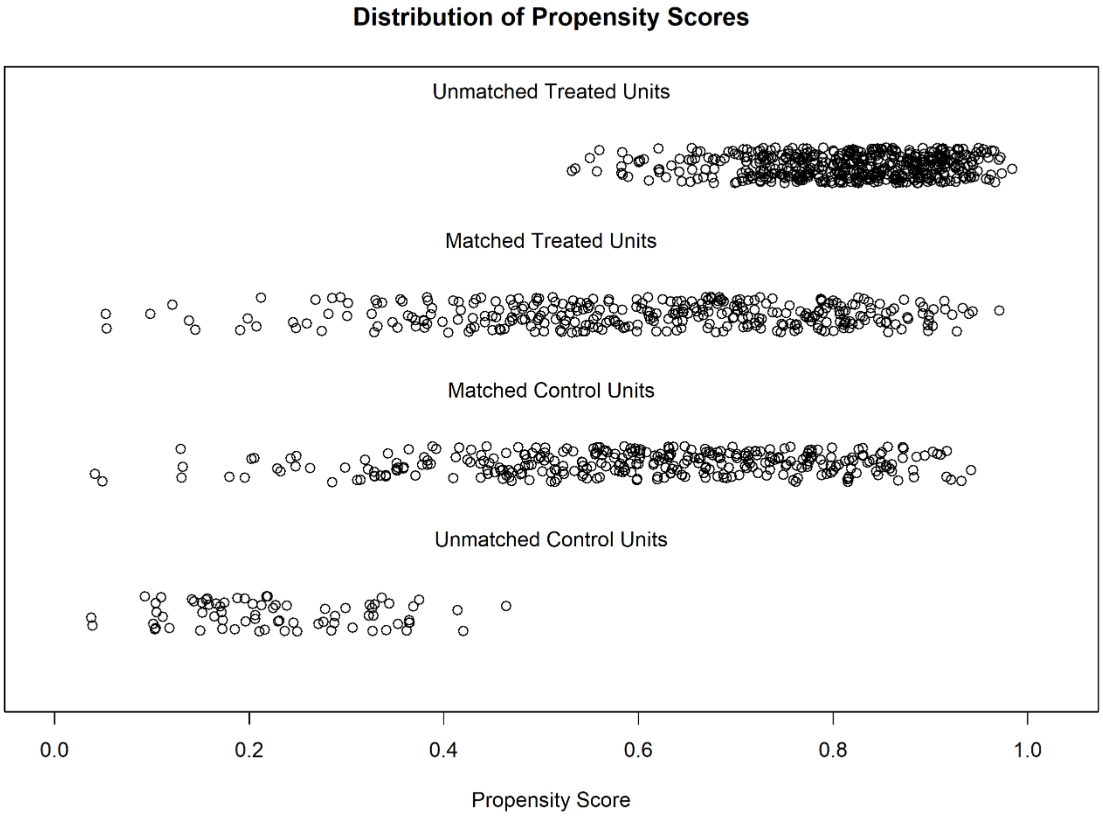

Figure S1. Distribution of propensity scores between treated (invasive managed) and control (conservative managed) patients.

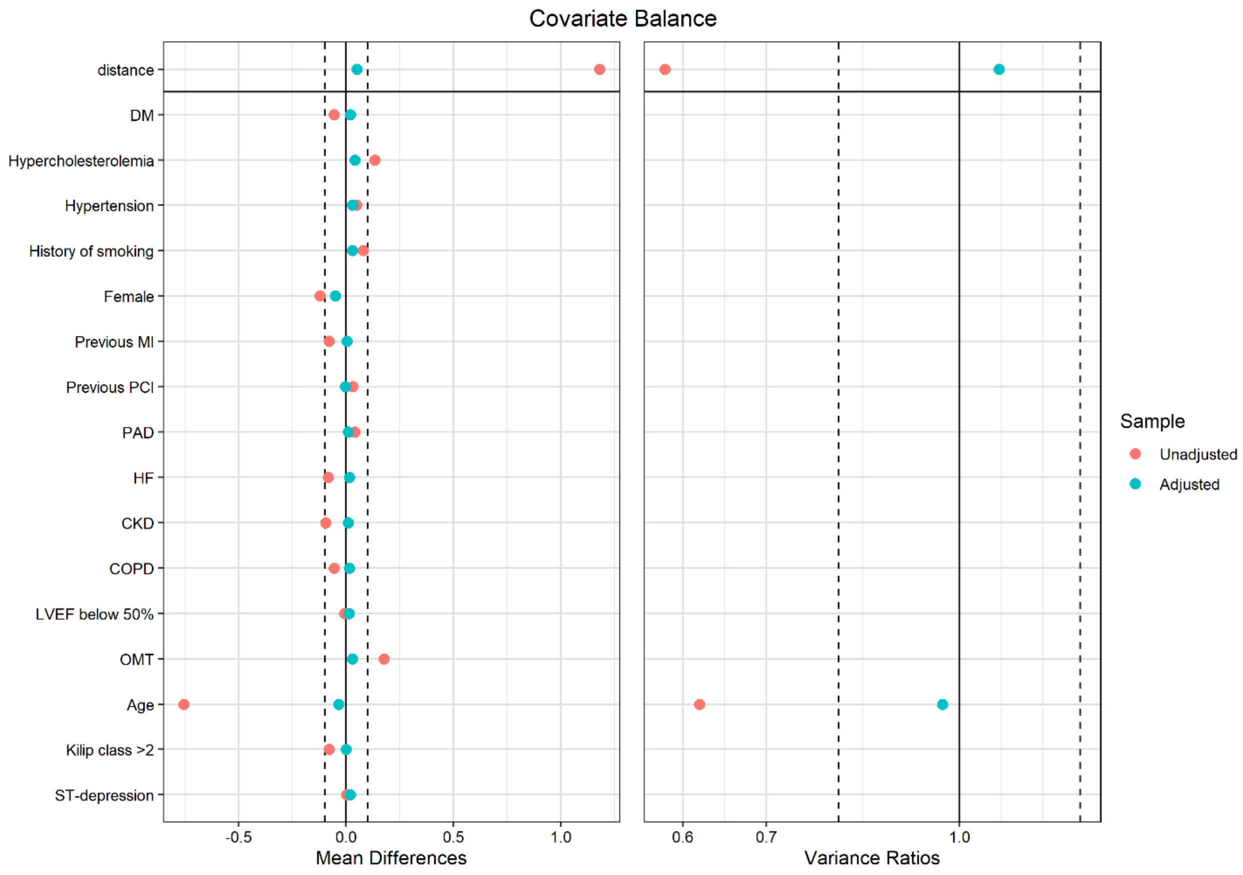

Figure S2. Balance for each covariates used in the propensity score matching analysis.
